# Multiresolution comparison of fetal real-time and cine magnetic resonance imaging at 0.55T

**DOI:** 10.1016/j.jocmr.2025.101856

**Published:** 2025-02-07

**Authors:** Datta Singh Goolaub, Ye Tian, Joshua F.P. van Amerom, John Wood, Jon Detterich, Krishna S. Nayak, Christopher K. Macgowan

**Affiliations:** a Translational Medicine, The Hospital for Sick Children, Toronto, Ontario, Canada; b Ming Hsieh Department of Electrical and Computer Engineering, Viterbi School of Engineering, University of Southern California, Los Angeles, California, USA; c Division of Cardiology, Children's Hospital Los Angeles, Los Angeles, California, USA; d Department of Medical Biophysics, University of Toronto, Toronto, Ontario, Canada

**Keywords:** Fetal, Low-field MRI, Cardiac, Cine imaging, Motion correction

## Abstract

**Background:**

Dynamic fetal cardiovascular MRI (CMR) enables visualization of moving structures to assess congenital heart disease and plan treatment. Low field MRI systems can provide more comfortable platforms for fetal CMR. Here, we demonstrate the feasibility and utility of motion corrected fetal cardiac cine CMR and compare it with real-time CMR at multiple spatial resolutions at 0.55 T.

**Methods:**

Ten human pregnancies were scanned at 0.55T on a derated MAGNETOM Aera (Siemens Healthineers, Erlangen, Germany) with spiral steady-state free precession imaging. Real-time images were reconstructed and used for motion correction and fetal cardiac gating followed by cine reconstructions. The signal-to-noise ratio (SNR), image quality, blood-to-myocardium contrast, and contrast-to-noise ratio (CNR) from real-time and cine reconstructions were compared. The effect of acceleration on cine accuracy was assessed by retrospectively undersampling the data and measuring the reconstruction error with the normalized root-mean-squared difference (NRMSD) in five fetuses. Reproducibility of the measurements was assessed by reconstructing cines from two independent windows of data and computing the NRMSD relative to the reference image in five fetuses.

**Results:**

The SNR, CNR, and image quality were better for cines than their corresponding real-time reconstructions. The blood-to-myocardium contrast had no significant difference between real-time and cine reconstructions. With finer spatial resolution, real-time images degraded, and cardiac structures were less conspicuous. NRMSD in cines decreased with increasing scan times across all resolutions (NRMSD = 10 ± 2% for 7 s scan duration). Good consistency (NRMSD = 11 ± 3%) was achieved between independent reconstruction windows.

**Conclusion:**

While this study was performed on an experimental scanner (derated; not commercially available), we have shown that fetal cine CMR is feasible at 0.55T and provides high-quality fetal cardiac images at high spatiotemporal resolutions.

## Graphical abstract

**Figure:**
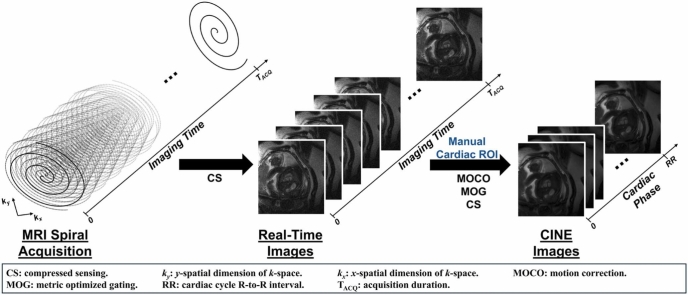

## 1 Introduction

Fetal cardiovascular magnetic resonance (CMR) imaging methods have recently been developed to assess fetal pathologies [1], [2], [3]. Using these methods, dynamic imaging, in the form of real-time images or cardiac-gated time-series images (CINEs), has been made possible allowing for visualizing of moving structures and assessing cardiac function [1], [4], [5]. In turn, this knowledge allows better planning of in-utero or postnatal treatment.

Fetal CMR faces several challenges. Fetal cardiac structures are small, and a high spatial resolution is needed to resolve cardiac malformations [6], [7]. Imaging at high spatial resolution requires greater *k*-space sampling such that imaging time with conventional magnetic resonance imaging (MRI) methods would be long. Accelerated imaging, such as compressed sensing (CS), is needed to address the long imaging times. Moreover, sporadic fetal gross motion and quasiperiodic maternal respiratory motion corrupt MRI acquisitions such that there is a need to quantify and compensate for the different types of motion [8]. Furthermore, fetal heart rates are high such that a high temporal resolution is needed to resolve the dynamic structures [9]. Longer acquisitions are then required to adequately sample the temporal domain of *k*-space. Additionally, there is a lack of conventional external gating method for fetal CINE CMR [10]. Gating is required to synchronize the acquisition with the cardiac phase to generate image series for representative cardiac cycles. One self-gating method that is useful to address this issue is metric optimized gating (MOG) which iteratively bins the acquired data based on an evolutionary heart rate model to generate a CINE that minimizes the image entropy [11]. Last, maternal comfort during imaging is limited by patient position, acoustic noise, and scanner bore size [12].

Recently developed low-field, wider-bore MRI systems (0.55T, 80 cm) with lower acoustic noise have potential for providing a more comfortable and accessible platform for fetal CMR [13]. Low-field MRI systems are suited for steady-state free precession (SSFP) imaging since they exhibit lower field inhomogeneity and, consequently, reduced banding artifacts from off-resonance effects [14]. Combined with efficient *k*-space sampling schemes, such as spiral readouts, and accelerated imaging, low-field MRI systems can also address requirements of high spatiotemporal resolutions for dynamic CMR [15], [16]. There are two avenues for dynamic CMR: real-time reconstructions—they allow for dynamic visualization of cardiac function and anatomy but are limited by low signal-to-noise ratio (SNR) at high spatiotemporal resolutions [17]—and CINE reconstructions—they combine data acquired over many heartbeats to yield dynamic visualization with high SNR but are limited by motion corruption and the need for a cardiac gating signal. The goal of this work was to demonstrate and compare real-time and CINE reconstructions of spiral SSFP images of the human fetal heart at 0.55T. Here, we apply motion correction (MOCO) with a CINE reconstruction framework for fetal spiral SSFP, previously developed for radial imaging at 1.5T [18], and demonstrate its utility for fetal CMR at high spatiotemporal resolution with spiral imaging at 0.55T.

## 2 Methods

### 2.1 Fetal cardiac MRI data acquisition

Ten pregnant women with healthy pregnancies, confirmed with ultrasound, were recruited for this study. The women (gestational age 28–34 weeks [mean = 32 ± 3 weeks, median = 32 weeks, interquartile range = 6 weeks], denoted by fetus 1–10) were imaged under free breathing conditions using a whole-body 0.55T prototype scanner. This was a derated 1.5T MAGNETOM Aera (Siemens Healthineers, Erlangen, Germany) equipped with high-performance shielded gradients (45 mT/m amplitude, 200 T/m/s slew rate). The study was approved by the Institutional Review Board and written informed consent was provided for all participants. Acquisitions were performed with spiral SSFP MRI using the following parameters: field-of-view = 240 × 240 mm^2^, slice thickness = 4 mm, spatial resolutions = 1.7 × 1.7 mm^2^, 1.5 × 1.5 mm^2^, and 1.0 × 1.0 mm^2^, spiral-out trajectory, imaging time = 11–19 s, spiral interleaves = 63, echo time = 0.8–0.9 ms, repetition time = 5.3–5.7 ms, flip angle = 90°, trajectory = pseudo golden angle (repeated after 144 arms), and trajectory correction with gradient impulse response function [19]. Single-slice acquisitions were performed to capture four-chamber views (in five fetuses) and short-axis views (in five fetuses).

### 2.2 Reconstruction of fetal cardiac MRI data

**Fig. 1:**
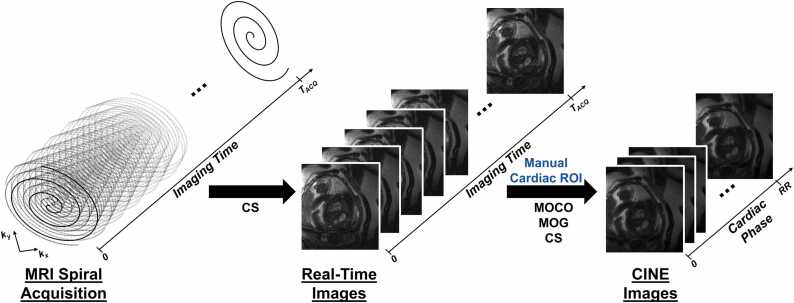
CINE reconstruction pipeline for fetal spiral SSFP MRI acquisitions. Following the acquisition of spiral data, real-time reconstructions are performed using compressed sensing. A manual region of interest is drawn to include the fetal heart. These real-time series are then processed to quantify gross motion and to extract the fetal cardiac gating signal, respectively. Finally, the data are motion corrected and cardiac gated resulting in high-quality output CINE reconstructions, again using compressed sensing. *CS* compressed sensing, *k*_*y*_*y*-spatial dimension of *k*-space, *k*_*x*_*x*-spatial dimension of *k*-space, *MOCO* motion correction, *MOG* metric optimized gating, *RR* cardiac cycle R-to-R interval, *T_ACQ_* acquisition duration, *CINE* cardiac-gated time-series images, *ROI* region of interest, *MRI* magnetic resonance imaging, *SSFP* steady-state free precession.

All reconstructions were performed using MATLAB (MathWorks, Natick, Massachusetts) on a computer with specifications: 64 GB random access memory and Intel® Core™ i9-9900k (3.60 GHz, 8 cores) central processing unit. Real-time reconstructions were first performed with CS (temporal finite difference = 0.08, 20 iterations) using 15 arms with 10 arms shared between frames (interpolated temporal resolution of ∼29 ms) using framework from [20] (Fig. 1). A region of interest (ROI) is drawn manually around the fetal heart for MOCO and MOG (an example ROI for fetus 7 for the 1.7 mm real-time reconstruction is shown in Supplementary Fig. 1). Sources of motion arising from the fetal anatomy were then resolved. Data rejection from through-plane motion was performed based on mutual information (MI) between real-time frames followed by translational MOCO [21], [22]. Motion-corrected real-times were then used to derive the variable fetal heart rate using MOG [11]. The gated and motion-corrected *k*-space was then reconstructed into a CINE (20 cardiac phases, temporal resolution ∼22 ms, 50 iterations) using CS (temporal finite difference = 0.02).

### 2.3 Comparison between real-time and CINE fetal reconstructions

Three regions of interest were drawn in real-time and CINE reconstructions. One ROI was drawn near the edge of the reconstructed field-of-view to measure the standard deviation of the noise. One ROI was drawn in the blood pool in the fetal heart to obtain the mean blood signal. One ROI was drawn in the myocardium (interventricular septum in eight fetuses; left ventricular free wall in two fetuses) to obtain the mean myocardial signal. Example ROIs in fetus 7 for the 1.7 mm resolution reconstructions are shown in Supplementary Fig. 2. Fetal real-time and CINE reconstructions were compared in four ways. First, the SNR from the blood pool in the fetal heart at the end of diastole was measured. SNR was computed using the ratio of the mean blood pool signal to the standard deviation of the measured noise signal. Second, the contrast between the blood and myocardium in diastole was computed. Third, the contrast-to-noise ratio (CNR) was also computed between the blood and myocardium in diastole. Fourth, the image perception quality of the fetal heart was quantified using a perception-based image quality evaluator (PIQUE, where lower values denote better image quality) which provides a no-reference image quality score [23]. This PIQUE metric captures the image quality by leveraging human perception of distortions and how the quality of small image patches affects the perception of the whole image. A two-sample Student’s *t*-test, with a significance value set to 0.05, was performed to compare each metric obtained from the real-time reconstructions and their corresponding CINE reconstructions.

### 2.4 Acceleration in fetal cardiac CINE reconstructions

To assess the effect of acceleration on CINE image quality, the 1.7, 1.5, and 1.0 mm resolution datasets were also reconstructed into CINEs using increasing number of arms (250–2500 at increments of 250, where acquiring 250 arms required ∼1.425 s). Five of the 10 datasets provided sufficient data, after rejecting data from gross motion, for this analysis. The CINE reconstruction from all available data in each dataset was used as a reference. The normalized root-mean-squared difference (NRMSD) between the reference and the accelerated reconstructions was quantified across the fetal anatomy [18].

### 2.5 Consistency in fetal cardiac CINE reconstructions

Using the same 5 datasets from above, the reproducibility of the acquisitions was analyzed by dividing the acquired data into 2 windows of 1250 independent spiral arms (∼7 s of scan time) and then performing CINE reconstructions from each (with S1 denoting data from the first window and S2 denoting data from the second window). The NRMSD between each reconstruction and the reference, from above, was computed across the fetal anatomy to assess consistency.

## 3 Results

### 3.1 Reconstruction of fetal cardiac MRI data

**Fig. 2:**
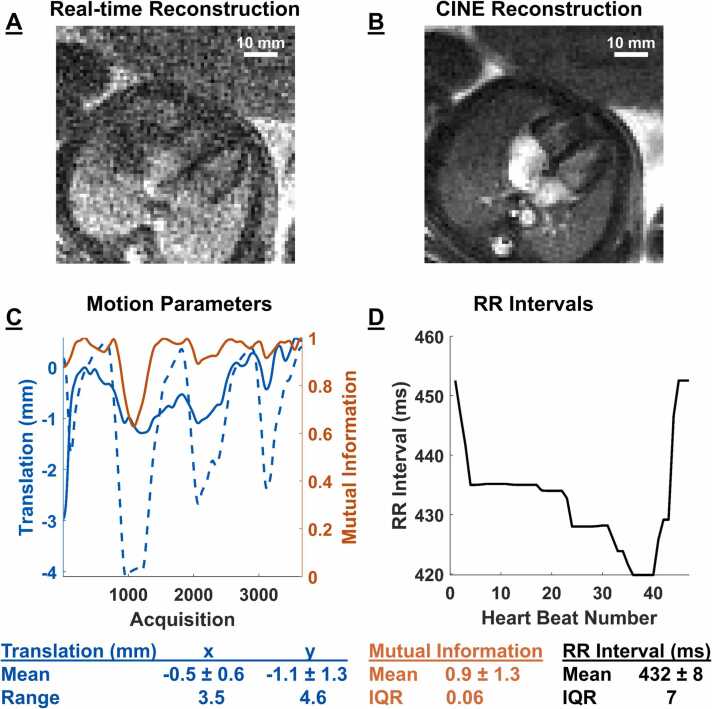
Representative fetal cardiac reconstructions and intermediate parameters. (A) Real-time and (B) CINE reconstructions from spiral acquisitions at 1.0 mm resolution in fetus 7. (C) Motion parameters obtained by tracking the heart in real-time frames. Interpolated translational displacements are in blue and mutual information between a given frame with respect to all other frames is in red. In this example, the translational range was [x, y] = [3.5, 4.6] mm and the mutual information between the real-time frames was 0.9 ± 1.3. (D) Measured beat-to-beat, RR, intervals (432 ± 8 ms) from real-time images over the duration of the scan. *CINE* cardiac-gated time-series images, *RR* cardiac cycle R-to-R interval, *IQR* interquartile range.

Real-time and CINE reconstructions were successful in all cases. Real-time reconstructions took approximately 45 min to complete 20 iterations. CINE reconstructions took approximately 10 min to complete 50 iterations. From MOCO using the real-time reconstructions, 10 ± 15% (for e.g. 399/3760 at 1.7 mm in fetus 10), with an interquartile range of 7%, of the data was rejected from each fetal acquisition owing to gross movement (with one case needing 85% (2319/2721) rejection). The range of the measured in-plane [x, y] translational motion was [4.1, 5.8] mm. The mean beat-to-beat (RR) interval across all fetuses was 433 ± 24 ms. Fig. 2 depicts representative 1.0 mm resolution real-time and CINE fetal cardiac reconstructions in fetus 7 along with a summary of motion parameters (translation range = [3.5, 4.6] mm, MI = 0.9 ± 1.3) and derived fetal RR intervals (432 ± 8 ms).

### 3.2 Comparison between real-time and CINE fetal reconstructions

**Fig. 3:**
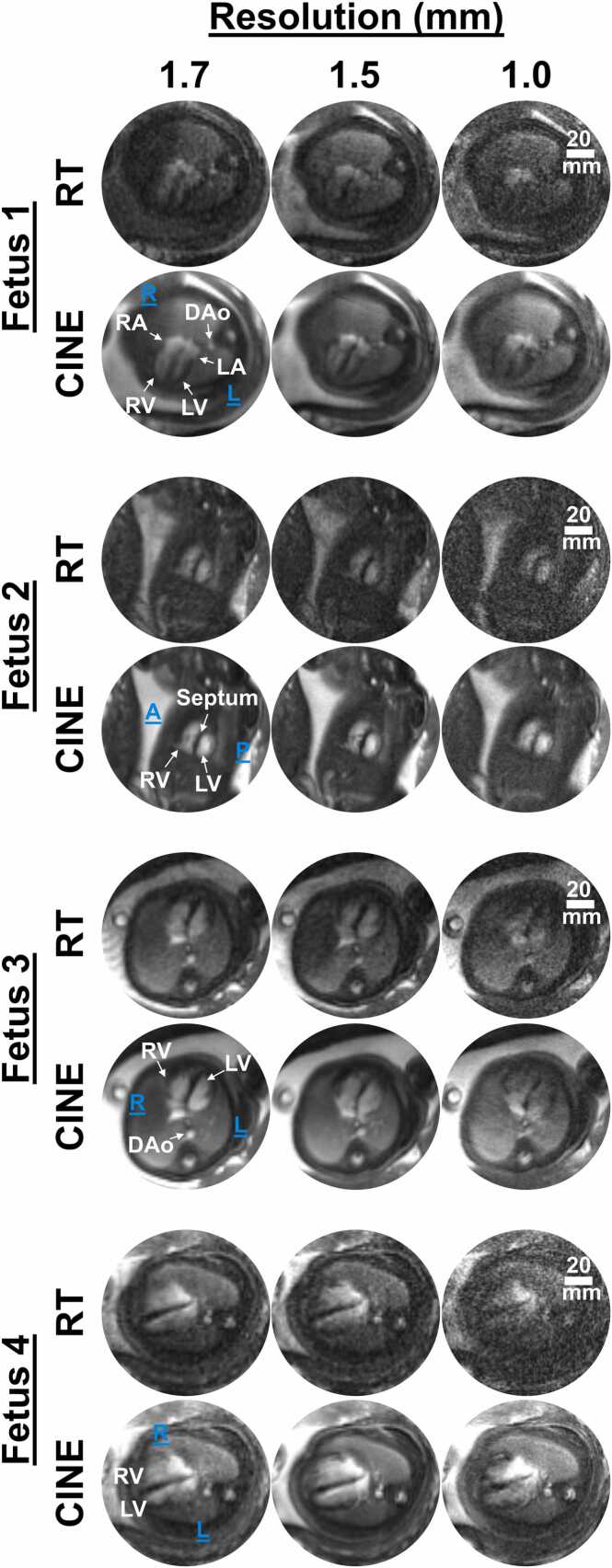
Real-time and CINE reconstructions at 1.7, 1.5, and 1.0 mm resolutions. Four-chamber view (fetus 1, 3, and 4). Short-axis view (fetus 2). Decrease in SNR as the spatial resolution becomes finer is observed. Improvement in SNR in CINE relative to real-time reconstructions is noticeable with cardiac structures becoming more conspicuous. For fetus 4, 85% (2319/2721) of the data was rejected due to through-plane motion in the 1.7 mm case. *SNR* signal-to-noise ratio, Direction: *A* anterior, *L* left, *R* right, *P* posterior, Anatomy: *DAo* descending aorta, *LA* left atrium, *LV* left ventricle, *RA* right atrium, *RV* right ventricle, *CINE* cardiac-gated time-series images, *RT* real time.

**Fig. 4:**
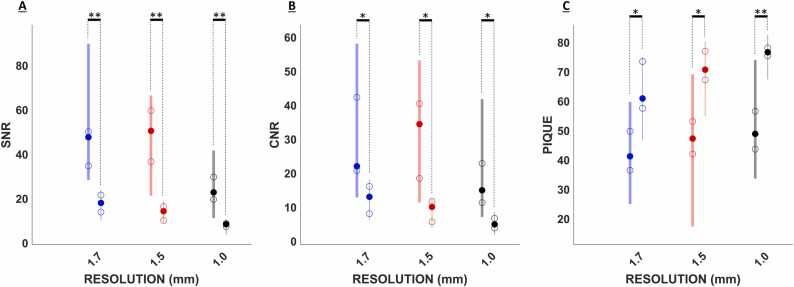
SNR, CNR, and PIQUE comparisons between CINE and real-time reconstructions. SNR (A), CNR (B), and PIQUE (C) for CINE (thick lines) and real-time (thin lines) reconstructions are depicted at 1.7 mm (blue), 1.5 mm (red), and 1.0 mm (black). Significance level: *p < 0.05 and **p < 10^−4^. Median (solid circle), upper/lower quartile (empty circle), and range (vertical line) are shown for each metric. SNR, CNR, and PIQUE measurements show significant differences between real-time and CINE reconstructions at all resolutions. The worst SNR, CNR, and PIQUE measurements were observed in 1.0 mm real-time reconstructions. *CNR* contrast-to-noise ratio, *PIQUE* perception-based image quality evaluator metric, *SNR* signal-to-noise ratio, *CINE* cardiac-gated time-series images.

Fig. 3 depicts a comparison between real-time and CINE fetal cardiac reconstructions from four fetuses (fetuses 1–4) and Table 1 summarizes the corresponding intermediate reconstruction results and image quality metrics. Dynamic versions of these reconstructions are provided in Video 1. Real-time reconstructions depict the dynamic fetal heart along with translational motion from maternal breathing and gross motion from fetal movement. With finer spatial resolution, the quality of the real-time reconstructions degraded, and cardiac details became less conspicuous. This was reflected by the quantitative measures of SNR, CNR, and PIQUE (Figs. 3 and 4). The change in contrast was not statistically significant. CINE reconstructions depict the dynamic fetal heart with in-plane fetal MOCO. Like real-time reconstructions, with finer spatial resolution the SNR, CNR, and PIQUE metrics for CINE reconstructions showed image degradation (Figs. 3 and 4) with the change in contrast not statistically significant.

**Table 1:** The measured SNR, CNR, and PIQUE in the reconstructions along with the range of translation offsets, mutual information between retained data, RR intervals, and total number of spiral arms used for CINE reconstructions are reported from fetus 1–4 at 1.0, 1.5, and 1.7 mm spatial resolutions are summarized.

Fetus	Metrics	1.7 mm	1.5 mm	1.0 mm
Fetus 1	SNR RT/CINE	14/34	16/47	7/20
	CNR RT/CINE	8/22	12/32	6/15
	PIQUE RT/CINE	75/50	57/17	78/56
	MOCO (mm)	[5.7, 5.3]	[5.1, 5.4]	[3.2, 4.0]
	MOG (ms)	436±10	425±19	445±9
	MI	0.82±0.15	0.92±0.05	0.94±0.03
	CINE arms	3124	3398	3328
Fetus 2	SNR RT/CINE	24/35	19/47	8/20
	CNR RT/CINE	16/56	12/40	4/16
	PIQUE RT/CINE	48/36	67/47	76/51
	MOCO (mm)	[4.4, 5.4]	[1.8, 5.0]	[4.1, 4.6]
	MOG (ms)	429±12	432±9	434±13
	MI	0.95±0.07	0.94±0.04	0.94±0.06
	CINE arms	3248	3325	2963
Fetus 3	SNR RT/CINE	21/75	15/39	9/20
	CNR RT/CINE	15/58	11/21	5/11
	PIQUE RT/CINE	58/56	55/37	76/43
	MOCO (mm)	[1.3, 2.1]	[1.4, 3.2]	[4.0, 3.9]
	MOG (ms)	427±11	409±11	427±10
	MI	0.93±0.05	0.93±0.06	0.97±0.02
	CINE arms	3494	3133	3195
Fetus 4	SNR RT/CINE	22/44	16/56	10/23
	CNR RT/CINE	15/22	11/36	7/15
	PIQUE RT/CINE	58/39	71/49	67/34
	MOCO (mm)	[4.5, 9.1]	[3.9, 5.1]	[2.5, 3.0]
	MOG (ms)	406±11	436±9	443±8
	MI	0.93±0.05	0.92±0.09	0.97±0.02
	CINE arms	402	2292	2577

Data are measured numerical values, [range along *x*-direction, range along *y*-direction], or mean ± standard deviation.

*CINE* cardiac-gated time-series images, *CNR* contrast-to-noise ratio, *MOCO* range of translation offsets, *MI* mutual information between retained data, *MOG* RR intervals, *PIQUE* perception-based image quality evaluator, *RT* real-time image series, *SNR* signal-to-noise ratio

Supplementary material related to this article can be found online at doi:10.1016/j.jocmr.2025.101856.

The following is the Supplementary material related to this article Video S1..

Video S1Side-by-side comparison between real-time and CINE reconstructions at 1.7, 1.5, and 1.0 mm resolutions in four fetuses. Four-chamber view (A, C, and D). Short-axis view (B). Improvement in SNR in CINE relative to real-time images is noticeable with cardiac structures becoming more conspicuous. Direction: *A* anterior, *L* left, *R* right, *P* posterior, Anatomy: *DAo* descending aorta, *LA* left atrium, *LV* left ventricle, *RA* right atrium, *RV* right ventricle

Comparing real-time versus CINE reconstructions, SNR, CNR, and image quality denoted by PIQUE were significantly higher (*p* < 0.05) for CINE reconstructions at all resolutions (Fig. 4). Across all scans, the worst SNR, CNR, and PIQUE measurements were observed in 1.0 mm real-time reconstructions. The SNRs of CINEs were higher than their corresponding real-time reconstructions at all resolutions by the following factors: 1.7 mm = 2.8 ± 0.6, 1.5 mm = 3.4 ± 1.1, and 1.0 mm = 3.1 ± 0.8. The CINEs at 1.0 mm showed better SNR than real-time reconstructions at all imaged spatial resolutions. The CNRs of CINEs were also higher than their corresponding real-time reconstructions by the following factors: 1.7 mm = 2.5 ± 0.8, 1.5 mm = 3.6 ± 1.4, and 1.0 mm = 3.4 ± 1.3. The PIQUEs of CINEs denoted better image quality than the corresponding real-time reconstructions with the PIQUE measurements differing by the following amounts: 1.7 mm = 19 ± 10, 1.5 mm = 23 ± 7, and 1.0 mm = 25 ± 8. There was no significant difference in blood-to-myocardium contrast measurements between CINE and real-time reconstructions at all resolutions (1.7 mm: *p* = 0.88, 1.5 mm: *p* = 0.10, 1.0 mm: *p* = 0.16). The SNR and PIQUE measurements were generally best for the 1.7 mm CINE reconstructions, with the 1.5 mm cases showing similar medians.

The 1.0 mm real-time and CINE reconstructions depict noticeable flow enhancements which are not visible in the 1.5 mm and 1.7 mm reconstructions. These observations are most evident in the four-chamber views which show prominent mitral valve inflow effects (Video 1A, C, and D). The 1.0 mm reconstructions also depict the septal geometry slightly better through sharper edges compared to the 1.5 and 1.7 mm which show relatively more blurred and rounded edges.

### 3.3 Acceleration in fetal cardiac CINE reconstructions

**Fig. 5:**
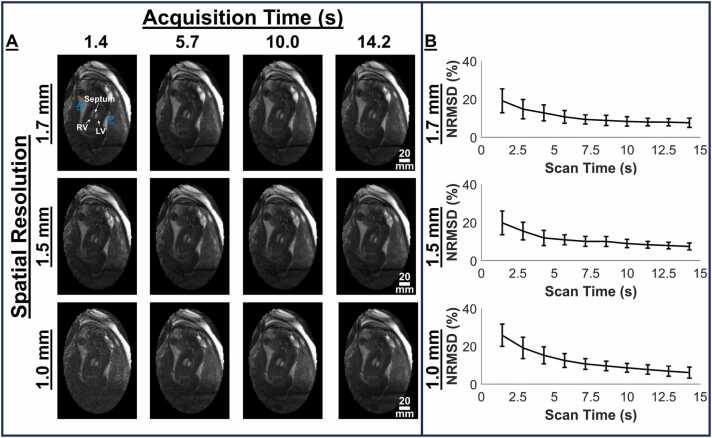
Effect of scan time on image quality of CINE reconstruction. (A) Representative reconstructed CINEs for one fetal case (fetus 2) at resolutions of 1.7, 1.5, and 1.0 mm using data from acquisition durations 1.4, 5.7, 10.0, and 14.2 s, respectively. (B) Image error given by NRMSD as a function of scan time at the corresponding spatial resolutions. NRMSD decreases monotonically with increasing scan time, with 10% error achieved by approximately 7 s scan time for all resolutions. *NRMSD* normalized mean-root-squared difference, Direction: *A* anterior, *P* posterior, Anatomy: *LV* left ventricle, *RV* right ventricle, *CINE* cardiac-gated time-series images.

Fig. 5 depicts the effects of acceleration on image quality on a short-axis view scan (fetus 2) along with the measured NRMSD in all tested cases at all imaged spatial resolutions. Five fetal cases (fetuses 2, 3, 8, 9, and 10) were used which had sufficient data for analysis after compensating for gross fetal motion through data rejection. Dynamic versions of these reconstructions are provided in Video 2. With increasing scan times, the image qualities improved (Fig. 5A). Undersampling artifacts faded, and SNR increased, making fetal cardiac structures more conspicuous. NRMSD errors, relative to the reference, decreased monotonically with increasing scan times for all resolutions (Fig. 5B). Assuming an acceptable NRMSD of 10%, CINE reconstructions could be achieved with scan times as low as approximately 7 s.

Supplementary material related to this article can be found online at doi:10.1016/j.jocmr.2025.101856.

The following is the Supplementary material related to this article Video S2..

Video S2Effect of scan time used for CINE reconstruction on image quality at 1.7, 1.5, and 1.0 mm resolutions in fetus 2. Image quality improves with increasing scan time with improvement in signal-to-noise ratio and reduction of undersampling artifacts. Direction: *A* anterior, *P* posterior, Anatomy: *LV* left ventricle, *RV* right ventricle

### 3.4 Consistency in fetal cardiac CINE reconstructions

**Fig. 6:**
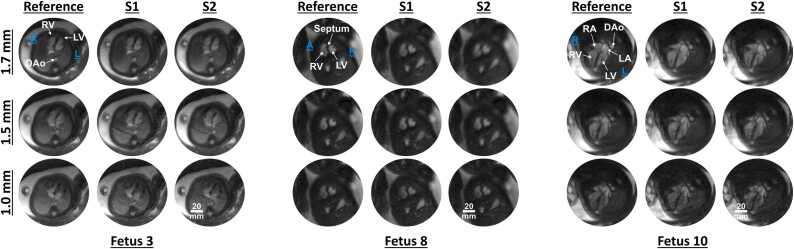
Reproducibility study at 1.7, 1.5, and 1.0 mm spatial resolutions. S1 and S2 depict the reconstructed CINEs using 7 s of data and Reference depicts a CINE reconstruction obtained by using all data. Good qualitative consistency is observed between S1 and S2 relative to the reference images. Residual streaking in S1 and S2 is observed in some cases owing to large gaps in *k*-space (clustering) corresponding to certain fetal heart rates. S2 for 1.7 mm in fetus 8 shows a blurred reconstruction relative to S1 and Reference owing to more pronounced fetal motion in the period during which data were acquired for S2. Direction: *A* anterior, *L* left, *R* right, *P* posterior, Anatomy: *DAo* descending aorta, *LA* left atrium, *LV* left ventricle, *RA* right atrium, *RV* right ventricle, *CINE* cardiac-gated time-series images.

Fig. 6 shows reconstructions in three fetuses (fetuses 3, 8, and 10) from two independent windows of data spanning 7 s of scan time. Dynamic versions of these reconstructions are provided in Video 3. The NRMSD between the reference and the reconstructed repeats was 11 ± 3%, 10 ± 3%, and 11 ± 4% for data scanned at 1.7, 1.5, and 1.0 mm, respectively. This depicts that the reconstructions exhibit consistency and repeatability. Residual streaking artifacts can be observed in the repeated reconstructions S1 and S2 in fetus 3 at 1.5 mm; however, these streaks are absent in the reference reconstruction which uses all scan data. This is because, in addition to a higher acceleration factor in S1 and S2, the cardiac-gated data being binned into the 20 cardiac phases exhibit clustering in *k*-space which is less prominent in the reference reconstruction. S2 in fetus 8 at 1.7 mm shows a blurred reconstruction relative to the reference and S1 reconstructions. This is because the fetal motion was greater during the acquisition of data used to reconstruct S2 [MI = 0.89 ± 0.03, range MI = 0.80–0.94, 13% (163/1250) data rejection]. With MOCO and data rejection, in the reference reconstruction, data from this period were rejected more relative to the data acquired during S1 [MI = 0.91 ± 0.02, range MI = 0.85–1, 2% (25/1250) data rejection] such that the final reference image appears sharper despite the presence of varying motion over the scan duration.

Supplementary material related to this article can be found online at doi:10.1016/j.jocmr.2025.101856.

The following is the Supplementary material related to this article Video S3..

Video S3Reproducibility study using windows of 7 s from acquired data at 1.7, 1.5, and 1.0 mm spatial resolutions from fetuses 3, 8, and 10. S1 and S2 depict the reconstructed CINEs using 7 s of data and Reference depicts a CINE reconstruction obtained by using all data. Direction: *A* anterior, *L* left, *R* right, *P* posterior, Anatomy: *DAo* descending aorta, *LA* left atrium, *LV* left ventricle, *RA* right atrium, *RV* right ventricle

## 4 Discussion

In this study, we compared real-time and motion-corrected CINE fetal CMR reconstructions of spiral SSFP data acquired at 0.55T performed at 1.7, 1.5, and 1.0 mm. Dynamic imaging of the fetal heart allows assessing cardiac malformations and cardiac function. Using CS, the data were first reconstructed into real-time series which showed cardiac motion, maternal breathing, and gross movement. Translational MOCO along with data rejection for through-plane motion were then performed. The fetal RR intervals were then computed with MOG. The resulting cardiac-gated, motion-corrected data were reconstructed in CINEs using CS. In doing so, we demonstrated the utility for fetal CMR at 0.55T. The low-field MRI system is also beneficial for fetal SSFP CMR since it provides higher maternal comfort, lower acoustic noise, and potentially reduced inhomogeneity-related artifacts relative to the currently used fetal MRI approaches [14], [24], [25].

While both real-time and CINE reconstructions provided dynamic reconstructions of the fetal heart at 0.55T, there was a ∼3-fold improvement in SNR and CNR when analyzing cardiac images in CINEs relative to real-time reconstructions. Since CINEs binned larger amounts of data in each reconstructed frame than real-time reconstructions, they benefited from greater *k*-space sampling and coverage. Moreover, the PIQUE metric was lower for the motion-corrected CINE reconstructions than for the corresponding real-time reconstructions, indicating superior image quality following amalgamation of the real-time data.

With the improved SNR and image quality in the motion-corrected CINE reconstructions, small fetal cardiac structures became more conspicuous. This was especially evident when scanning at very high spatial resolutions (1.0 mm) that push the SNR limits of real-time MRI at 0.55T. In turn, CINE reconstructions can allow resolving the very small cardiac structures in fetuses and assessing pathologies better. Clinically, these attributes are beneficial for accurate cardiac segmentation and analysis [5].

Although the SNRs of the real-time reconstructions were limited at the highest spatial resolutions explored in this study, it remained sufficient for MOCO and MOG. Gross MOCO was achieved by temporally smoothing the high temporal resolution real-time frames. As shown in our previous work, such smoothing can suppress cardiac motion which might otherwise bias gross motion tracking, while it also improves the SNR of the resulting frames to allow for more robust image registration and data rejection [18], [21]. Conversely, MOG required real-time reconstructions at the highest temporal resolution. MOG operates by binning the real-time frames into cardiac phases, based on a fetal heart-rate model, and then averaging them into representative frames with higher SNR. The resulting images are then used to minimize a gating metric. The metric is computed on the representative re-sorted CINE frames which have higher SNR than real-time frames. Hence, the optimization process exploits a sufficiently high SNR regime even when imaging was performed at 0.55T in this study.

There was no significant difference in blood-to-myocardium contrast between real-time and motion-corrected CINE reconstructions. This was because image contrast was mainly dictated by the low spatial frequency spiral *k*-space data which was densely sampled in both real-time and CINE reconstructions. In this study, 1.7 mm CINEs provided the best SNR and PIQUE measurements. However, this high SNR comes at the expense of increased partial volume effects. While the 1.0 mm CINEs provided good fetal cardiac visualizations and good inflow effects which are desirable contrast to investigate pathological conditions, they still suffered from relatively poor SNR. The 1.5 mm CINEs showed both acceptable SNR and image quality (less partial volume effects than 1.7 mm data). A viable candidate for optimal comprise between resolution and conspicuity for fetal CMR at 0.55T with the described protocol could be around 1.2 mm (which yields higher SNR than at 1.0 mm, and less partial volume effects than at 1.5 mm).

From the retrospective CINE acceleration experiment performed in this study, accuracy improved with longer scan times for all imaged resolutions. With 10% error being achieved on average at ∼7 s scan time for all resolutions, this represents the minimum scan duration needed to achieve reliable fetal cardiac images at 0.55T with spiral SSFP. To account for sporadic and uncontrollable fetal motion, 10% of the acquired data was generally rejected following each fetal acquisition; hence, a longer scan time may be required to achieve the high temporal resolution (22 ms) CINEs targeted in this study. The consistency experiment showed that the measurements within each fetal subject were repeatable at all imaged resolutions at 0.55T. The errors relative to the reference reconstruction in these two experiments can be attributed to three sources mainly: increased noise and undersampling artifacts, variation in amount of rejected data for MOCO, and variation in clustering of readouts in *k*-space for certain fetal heart rates during binning for CINE reconstruction, which cannot be fully compensated for with CS [26].

## 5 Limitations

Despite successfully demonstrating high-resolution CINE fetal CMR at 0.55T, this study had certain limitations. First, the prototype 0.55T scanner used in these experiments had high-performance gradient hardware which is not representative of commercial low-field MRI scanners at present (26 mT/m, 45 T/m/s). Weaker gradient performance can limit *k*-space sampling efficiency which influences repetition time or spiral arm length per repetition, thereby affecting the achievable temporal resolution of real-time reconstructions and thus potentially the quality of subsequent CINE reconstructions. Further exploration and optimization of these methods on commercial low-field systems are needed; however, initial studies have already demonstrated good agreement between commercial systems such as the Free.Max (Siemens Healthineers, Erlangen, Germany) and a prototype scanner like that used in the current study, albeit in other applications [27]. Second, reconstruction times were relatively long, impeding the clinical translation of these methods. In the future, more efficient reconstruction approaches, such as performant graphics processing unit approaches or machine learning-based reconstructions, will be explored to increase the clinical practicality of the studied methods. Third, the reconstruction pipeline was not fully automated. A manual region of interest containing the fetal heart had to be drawn to allow for MOCO and MOG. This step can limit the application of the reconstruction pipeline in fetal scans with large numbers of slices. Future work will look at incorporating deep learning segmentation methods to speed up the reconstruction pipeline, testing the approach with sequence adapted to low-field MRI gradient specifications, volumetric cardiac analysis, and investigate clinical metrics such as diagnostic sensitivity [28].

## 6 Conclusion

In conclusion, we have demonstrated the utility of fetal CINE SSFP CMR at 0.55T. This study shows that reliable fetal cardiac imaging can be achieved using low-field MRI systems, providing greater maternal comfort with low acoustic noise and larger bore size. With MOCO and retrospective gating, CINEs were able to provide high-quality reconstructions for spatiotemporal resolutions up to 1.0 and 20 ms.

## Funding

1. 10.13039/501100000024Canadian Institutes of Health Research
2. 10.13039/100006034University of Southern California (USC) Provost’s Strategic Direction for Research Award
3. Keck School of Medicine of USC Dean’s Pilot Grant

## Author contributions

**Datta Singh Goolaub:** Writing – review & editing, Writing – original draft, Visualization, Validation, Software, Methodology, Investigation, Formal analysis, Data curation, Conceptualization. **Ye Tian:** Writing – review & editing, Writing – original draft, Methodology, Data curation, Conceptualization. **Joshua F.P. van Amerom:** Writing – review & editing, Writing – original draft, Conceptualization. **John Wood:** Writing – review & editing, Writing – original draft, Funding acquisition, Conceptualization. **Jon Detterich:** Writing – review & editing, Writing – original draft, Conceptualization. **Krishna S. Nayak:** Writing – review & editing, Writing – original draft, Supervision, Funding acquisition, Conceptualization. **Christopher K. Macgowan:** Writing – review & editing, Writing – original draft, Supervision, Funding acquisition, Conceptualization.

## Ethics approval and consent

This study was approved by the Institutional Review Board and written informed consent was provided for each participant.

## Declaration of competing interests

None.

## Data Availability

https://github.com/datta-g/LowField-Fetal-CINE-CMR.
