# Supplementary material for: Multiresolution comparison of fetal real-time and cine magnetic resonance imaging at 0.55T
Source: J Cardiovasc Magn Reson. 2025 Feb 7;27(1):101856. doi: 10.1016/j.jocmr.2025.101856 (PMC11968261; doi:10.1016/j.jocmr.2025.101856)
Supplement: Supplementary file 4 — Supplementary material [file mmc1.pdf]

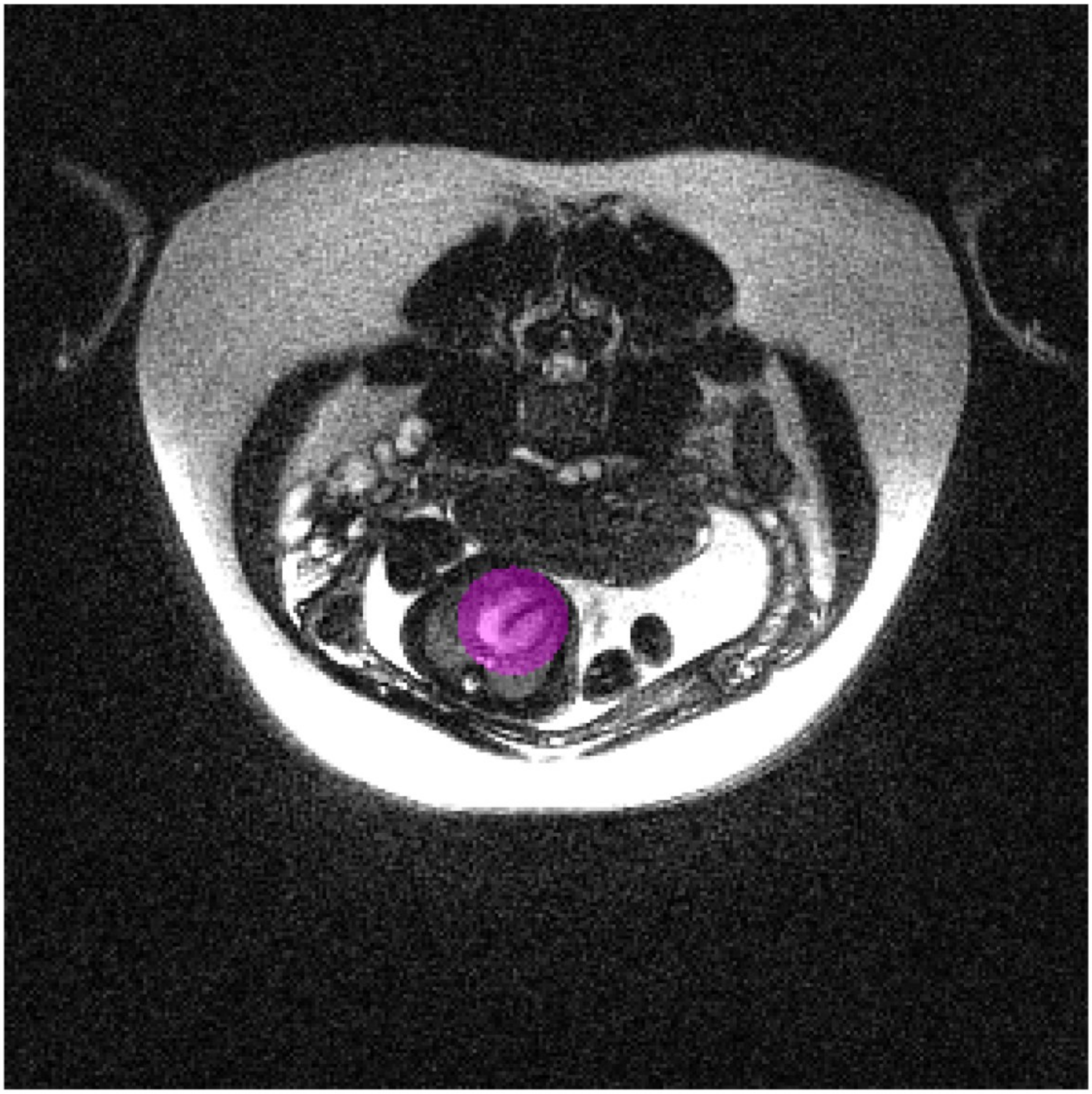

Supplementary Figure 1: Real-time frame in Fetus 7 at 1.7 mm resolution with the region of interest (ROI) for metric optimised gating (MOG) overlayed in magenta. The ROI comprises the fetal heart such that periodic motion of the cardiac structures can be used for optimization in MOG.
