# Supplementary material for: Multiresolution comparison of fetal real-time and cine magnetic resonance imaging at 0.55T
Source: J Cardiovasc Magn Reson. 2025 Feb 7;27(1):101856. doi: 10.1016/j.jocmr.2025.101856 (PMC11968261; doi:10.1016/j.jocmr.2025.101856)
Supplement: Supplementary file 5 — Supplementary material [file mmc2.pdf]

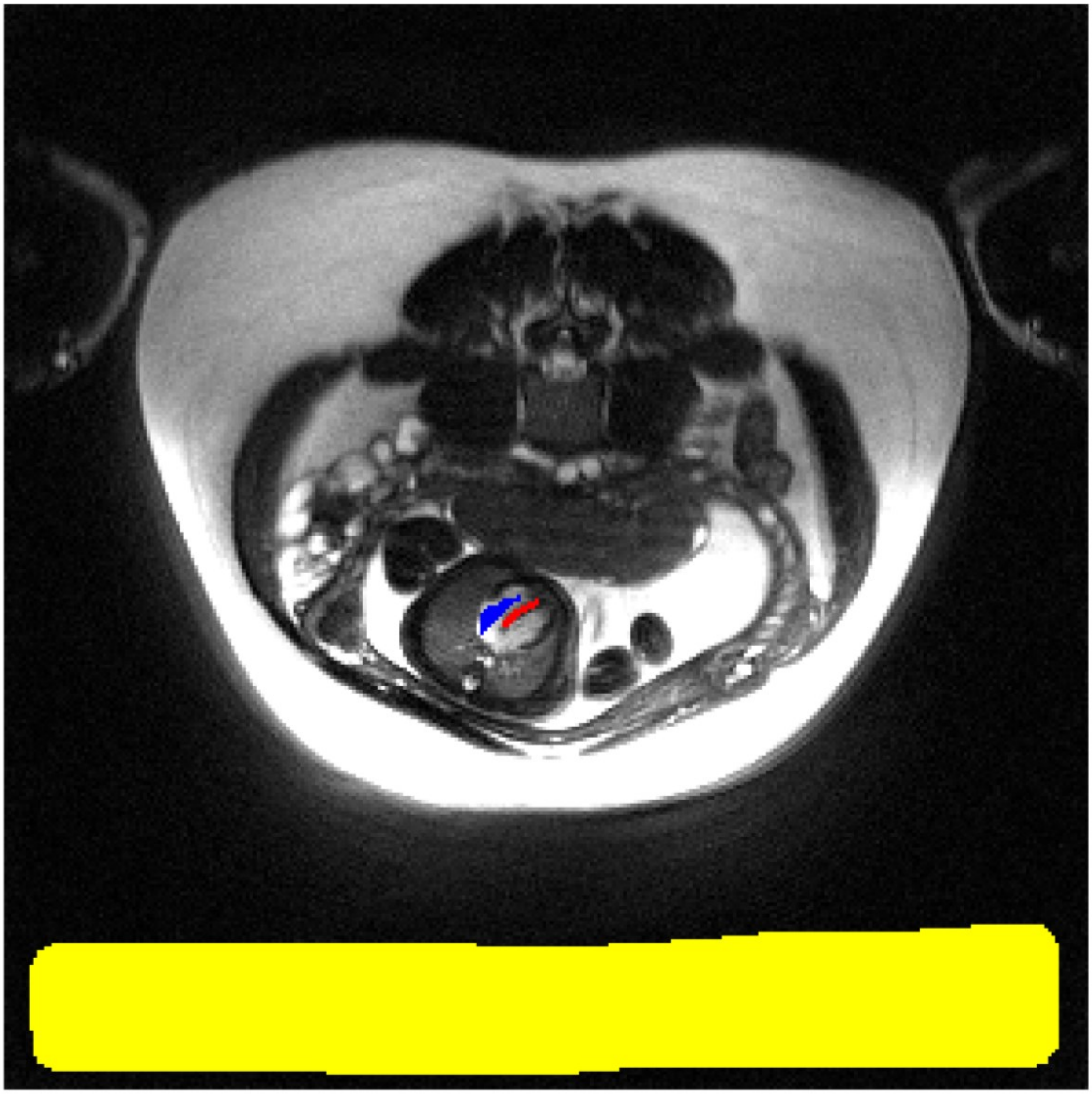

Supplementary Figure 2: Masks used to measure signal for metric computation in Fetus 7 at 1.7 mm resolution. Red: myocardium signal location. Blue: blood pool signal location. Yellow: noise signal measurement. For Fetus 7, the region of interest (ROI) for blood pool signal had an area of 118 mm^2 and for the myocardial signal had an area of 78 mm^2. Across all fetuses, the mean area for myocardial signal measurement was 54 ± 30 mm^2 and for blood pool signal measurement was 93 ± 56 mm^2. The ROI for measuring the standard deviation of the noise was placed near the edge of the reconstructed field-of-view.
